# Rejuvenated Brewer’s Spent Grain: EverVita Ingredients as Game-Changers in Fibre-Enriched Bread

**DOI:** 10.3390/foods10061162

**Published:** 2021-05-22

**Authors:** Aylin W. Sahin, Jonas Joachim Atzler, Daniel Valdeperez, Steffen Münch, Giacomo Cattaneo, Patrick O’Riordan, Elke K. Arendt

**Affiliations:** 1 School of Food and Nutritional Sciences, University College Cork, T12 YN60 Cork, Ireland; aylin.sahin@ucc.ie (A.W.S.); 117107223@umail.ucc.ie (J.J.A.); 2 EverGrain, LLC, One Busch Place, St. Louis, MO 63118, USA; Daniel.Valdeperez@Everingredients.com (D.V.); Steffen.Muench@Everingredients.com (S.M.); Giacomo.Cattaneo@everingredients.com (G.C.); 3 Global Innovation & Technology Centre, Anheuser-Busch InBev nv/sa, 3000 Leuven, Belgium; Patrick.ORiordan@ab-inbev.com; 4 APC Microbiome Institute, University College Cork, T12 K8AF Cork, Ireland

**Keywords:** BSG, plant protein, fibre fortification, glycaemic index, bread dough quality, gluten network, nutritional value

## Abstract

Brewer’s spent grain (BSG) is the main side-stream of brewing. BSG is a potential source for nutritionally enriched cereal products due to its high content of fibre and protein. Two novel ingredients originating from BSG, EverVita FIBRA (EVF) and EverVita PRO (EVP), were incorporated into bread in two addition levels to achieve a ‘source of fibre’ (3 g/100 g) and a ‘high in fibre’ (6 g/100 g) nutrition claim for the breads. The impact of those two ingredients on dough and bread quality as well as on nutritional value was investigated and compared to baker’s flour (C1) and wholemeal flour (C2) breads. The addition of EVF performed outstandingly well in the bread system achieving high specific volumes (3.72–4.66 mL/g), a soft crumb texture (4.77–9.03 N) and a crumb structure comparable with C1. Furthermore, EVF barely restricted gluten network development and did not influence dough rheology. EVP increased the dough resistance (+150%) compared to C1 which led to a lower specific volume (2.17–4.38 mL/g) and a harder crumb (6.25–36.36 N). However, EVP increased the nutritional value of the breads by increasing protein content (+36%) and protein quality by elevating the amount of indispensable amino acids. Furthermore, a decrease in predicted glycaemic index by 26% was achieved and microbial shelf life was extended by up to 3 days. Although both ingredients originated from the same BSG, their impact on bread characteristics and nutritional value varied. EVF and EVP can be considered as game-changers in the development of bread fortified with BSG, increasing nutritional value, and promoting sustainability.

## 1. Introduction

Due to increased environmental awareness, the sustainable use of side-streams generated within the food industry has become increasingly important, focused on eliminating waste and the continual use of resources. Various studies have been conducted to valorise food waste by recycling these materials into biofuels, enzymes and bioactive compounds [1]. From a nutritional perspective, some of these by-products provide a rich source of nutrients, including vitamins, minerals, protein, fibre and bioactive compounds and, therefore, may be useful for further food applications [2,3,4]. Brewer’s spent grain (BSG), the insoluble barley residue left after wort production, is the primary side-stream of brewing, representing 85% of the total by-products obtained [5]. The annual production is estimated to be 40 million tonnes worldwide, with 3.4 million tonnes generated in the European Union [6,7]. Currently, most BSG is used as a low-value animal feed with a cost of €35 per tonne [8].

BSG has huge potential to enhance the nutritional value of food due to its high content of fibre (30–50% *w/w*) and protein (19–30% *w/w*). The fibre is constituted of hemicellulose including arabinoxylans, cellulose and lignin [9]. The presence of arabinoxylans in the diet has been associated with various health benefits, including improved glycaemic control, reduced cholesterol, enhanced mineral absorption, faecal bulking and gut health [10,11]. The protein content of BSG is significant due to its composition of essential amino acids (~30%), especially lysine (~14.3%) [12].

The incorporation of BSG into cereal-based foods has been evaluated in several studies [12,13,14,15,16]. Bread is an important staple food worldwide due to its convenience, versatility, and low cost. In fact, bread has been reported as the main contributor to fibre intakes of Irish adults, although over 80% of the population are not reaching the European Food Safety Authority (EFSA) recommended intake of 25 g/d [17,18]. Therefore, the fibre enrichment of bread with BSG could be an adequate method to close the fibre gap. Several studies revealed the increase in the nutritional value of bread fortified with BSG showing an elevated protein and fibre content with high lysine concentrations and lower glycaemic index [19,20]. However, the replacement of wheat flour with BSG negatively impacts bread quality, resulting in a product with a lower volume, increased hardness, denser structure and a darker colour [12,16,21]. The addition of sourdough as a functional ingredient showed a positive impact on bread quality when BSG was added [22].

EverGrain (St. Louis, MO, USA) processed BSG and launched two ingredients, EverVita FIBRA and EverVita PRO, which differ in their protein and fibre content and particle size. A recent study characterised those ingredients and investigated their effect on pasta, resulting in outstanding product quality and increased nutritional value [23]. The current study reveals the impact of those two ingredients in two addition levels on bread dough quality including their effect on gluten network formation and starch pasting properties, as well as on final bread quality. Moreover, their effect on starch digestibility was determined using an in vitro starch digestibility model, and the protein quality in the bread based on the amount of indispensable amino acids was evaluated. A correlation analysis was conducted to provide a deep insight into the connected parameters, and two-way analysis of variance (ANOVA) revealed the influence of addition level or type of ingredient added and emphasised interaction effects of those two variables. 

## 2. Materials and Methods

### 2.1. Raw Materials

Baker’s flour and stoneground wholemeal flour supplied by Odlums (Dublin, Ireland), sunflower oil (Musgrave Wholesale Partners, Cork, Ireland), salt (Glacia British Salt Limited, Cheshire, UK), sugar (Nordzucker Ireland Ltd., Dublin, Ireland), baker’s instant active dried yeast (Bruggemean Puratos, Dilbeek, Belgium) and tap water were used in the bread production. The two ingredients, EverVita FIBRA (EVF) and EverVita PRO (EVP), rejuvenated brewer’s spent grain, were supplied by EverGrain, LLC (St. Louis, MO, USA). Chemicals used for analysis were purchased from Sigma-Aldrich (St. Louis, MO, USA), unless stated otherwise.

### 2.2. Nutritional Profile and Amino Acid Composition of Raw Ingredients

The compositional analysis of EVF and EVP were previously reported by Sahin et al., (2021) [23]. Baker’s flour and wholemeal flour were analysed by Concept Life Sciences Ltd., Manchester, UK using the following methods: moisture—gravimetric air-oven method at 130 °C; protein—modified Dumas method with nitrogen-to-protein conversion factor 6.25 (AOAC 197.992.15); fat—low resolution proton nuclear magnetic resonance (NMR) (MQC-23-35; Oxford instruments application note); dietary fibre—gravimetric method (AOAC 991.43); ash—removal of organic matter by oxidation (550 °C) (ISO 936:1998); sodium—flame photometry; carbohydrates—calculated by difference. The sugar profile including fructose, glucose, sucrose/maltose and maltotriose/raffinose were determined using a Dionex ICS-5000+ system (Thermo Fisher Scientific, Sunnyvale, CA, USA) equipped with an electrochemical detector. Samples were extracted in triplicate following the procedure reported by Hoehnel et al. (2020) [24] and analyses on the ICS-5000+ system were performed as stated by Ispiryan et al. (2019) [25].

Amino acid analysis was conducted by Mérieux NutriScience CHELAB S.r.l., Resana, Italy. After protein extraction and hydrolysis (independent hydrolysis procedure for analysis of tryptophan, sulphuric amino acids and remaining amino acids), amino acids were quantified using ionic chromatography with post-column ninhydrin derivatisation coupled with a fluorescence and an ultraviolet (UV) detector. The concentration of the amino acids is given as % based on protein.

### 2.3. Bread Dough Preparation Process

Bread doughs were produced using the ingredients according to the recipes illustrated in Table 1. Two different concentrations EverVita FIBRA and EverVita PRO were incorporated into a bread formulation by replacing baker’s flour. The addition levels were chosen to reach a ‘source of fibre’ (SF) and a ‘high in fibre’ (HF) claim according to European Union (EU) regulation [26].

Dry ingredients were pre-mixed for 1 min at minimum speed using a Kenwood Titanium Major KM020 mixer equipped with a hook attachment (Kenwood, Havant, UK) to ensure a homogeneous distribution, and instant active dried yeast was activated by its addition to water (25 °C) for 10 min. Yeast solution and sunflower oil were added to the dry ingredients and mixed at speed 1 for 1 min first, followed by a second mixing step at speed 2 for 7 min. This procedure was used for the baking process as well as for the evaluation of the fermentation quality of the bread doughs.

### 2.4. Fundamental Understanding of the Effect of EverVita Ingredients on Dough Properties

EverVita ingredients originate from BSG, which has been reported to impact bread dough quality negatively. Hence the effect of reinvented BSG-based ingredients on gluten network development, starch pasting properties as well as on dough rheology, extensibility and fermentability was investigated. Therefore, blends of baker’s flour and EVF/EVP according to the ratios given in Table 1 were analysed. As controls baker’s flour (control 1) and wholemeal flour (control 2) were chosen. 

#### 2.4.1. Gluten Network Formation and Starch Pasting Properties

The impact of EverVita ingredients on both gluten network and starch pasting was determined by the GlutoPeak and the Rapid Visco Analyser (RVA). For both measurements the solid part represents a mixing of baker’s flour and EverVita ingredient (EVF or EVP) in the proportion used for the bread baking process (Table 1).

The GlutoPeak^®^ (Brabender GmbH and Co. KG, Duisburg, Germany) was used to measure the gluten aggregation properties represented by the time of full aggregation (peak maximum time (PMT) in seconds (s)) and gluten network strength (torque maximum (TM) in Brabender units (BU)). 9 g of solids, based on 14% moisture, was added to 9 g of distilled water (36 °C) and the test was started using a rotation speed of 2750 rpm of the paddle.

Starch pasting properties during heating and cooking were determined using a Rapid Visco Analyser (Newport Scientific, Warriewood, Australia); 3 g of solids, based on 14% moisture, was added to 25 g distilled water in the metal sample cup and premixed briefly with the paddle to remove lumps. A constant shear rate of 160 rpm was applied during the measurement. The temperature profile used was equilibration at 50 °C for 1 min, followed by an increase to 95 °C with a heating rate of 0.2 °C/s, held at 95 °C for 162 s, cooled to 50 °C with a cooling rate of 0.2 °C/s, and held at 50 °C for 60 s. During the measurement the viscosity in centipoise (cP) was monitored resulting in a viscosity curve over time and peak viscosity (cP), breakdown viscosity (cP), trough viscosity (cP) and final viscosity (cP) were evaluated.

#### 2.4.2. Water Absorption and Dough Development during Mixing

The water content of the different formulations was adjusted using Farinograph-TS^®^ equipped with an automatic water dosing unit (Brabender GmbH and Co KG, Duisburg, Germany). Therefore, flour and EverVita ingredients were used in the ratio illustrated in Table 1. The target consistency of the doughs was 500 farinograph units (FU). After titration the optimal water content was used to determine the water absorption (WA; amount of water required to achieve a dough consistency of 500 FU), the dough development time (min) (DDT; time required to reach maximum consistency), the arrival of dough stability (min) (S1; length of time that dough consistency is held at 500 FU during mixing), and the mixing tolerance index (FU) (MTI; torque difference between maximum torque and torque five minutes after maximum) were evaluated. The optimal water content was used for preparing bread doughs.

#### 2.4.3. Extensibility and Resistance to Extension

The dough extensibility (E) in mm, resistance to extension (RE) in extensograph units (EU) and the ratio of resistance over extensibility (RE/E) in (EU/mm) were determined using Extensograph^®^ (Brabender GmbH and Co KG, Duisburg, Germany). The dough was prepared following the bread dough preparation process omitting yeast. 150 ± 0.5 g of dough (without addition of yeast) was moulded and placed into the proofing chamber. Analysis was performed after 90 min of proofing (time used for proofing during the baking process).

#### 2.4.4. Fermentation Quality

A rheofermentometer (Chopin, Villeneuve-la-Garenne, France) was used to measure gaseous release and dough rise during the proofing process. We prepared 300 g dough following the bread dough preparation process procedure mentioned before and transferred into the fermentation chamber (30 °C). A cylindrical weight of 1500 g was placed on the dough and the dough was fermented for 180 min. The maximum height of the dough (Hm) in mm, the time required to achieve maximum height (T1) in minutes, the total volume of carbon dioxide released by the dough (Vtot) in mL and the height of maximum gas formation (H’m) in mm were evaluated.

### 2.5. Baking Process

Dough was separated into 450 g pieces, moulded, transferred into a bread pan (dimensions: 15 × 9.5 × 9.7 cm) and placed into the proofing chamber (KOMA BV Sunriser, Roermond, The Netherlands) for 90 min set to 30 °C and 85% relative humidity. After proofing, the tins with the leavened doughs were transferred into a deck oven (MIWE Condo, Arnstein, Germany) (220 °C top temperature and 230 °C bottom temperature). 400 mL of steam was injected prior to loading. The breads were baked for 35 min, removed from the baking tins and left to cool for 120 min on a rack before analysis. Two breads per batch were analysed after baking and two loaves were packed in plastic bags and stored at 20 °C for five days to determine the staling rate. Three batches per bread type were evaluated.

### 2.6. Changes in Techno-Functional Properties of Bread Fortified with EverVita Ingredients

#### 2.6.1. Specific Volume

The specific volume was measured with a 3D laser scan using a Volscan Profiler 300 (Stable Micro Systems, Godalming, UK) and expressed as mL/g.

#### 2.6.2. Crumb Texture and Staling

The bread crumb texture was analysed using a TA-XT2i texture analyser (Stable Micro Systems, Godalming, UK) equipped with a 25 kg load cell. For analysis, the breads were sliced with a thickness of 25 mm. To imitate chewing activity, a two-compression test was carried out using a cylindric probe with a diameter of 35 mm, a test speed of 5 mm/s, a trigger force of 0.05 N and a compression of 40%. The crumb hardness in Newton (N) and chewiness (N) were evaluated 120 min after baking. Gumminess (N) showed the same values as chewiness and was not further considered in the manuscript. In addition, the hardness (N) was measured 120 h after baking to evaluate bread staling. Staling was expressed as staling rate as defined by Sahin, Axel, Zannini, and Arendt (2018) [27].

#### 2.6.3. Crumb Macro- and Microstructure

For the evaluation of crumb structure, a C-cell Imaging System (Calibre Control International Ltd., Warrington, UK) was used and slice area (mm^2^), number of cells and average cell diameter (mm) were evaluated. The crumb area for the evaluation of the number of cells and the cell diameter was the slice area. Furthermore, the microstructure of the crumb was visualised using a scanning electron microscope (SEM). Therefore, bread crumb was freeze-dried, immobilised, and coated with a layer of 25 mm of sputtered palladium-gold. The microstructure was observed using a working distance of 8 mm and micrographs were taken at an accelerating voltage of 5 kV. SEM Control User Interface software, Version 5.21 (JEOL Technics Ltd., Tokyo, Japan) was used. 

#### 2.6.4. Crust and Crumb Colour

A colorimeter (Minolta CR-331, Konica Minolta Holdings Inc., Osaka, Japan) was used to determine the bread colour. To evaluate the influence of EverVita ingredients on the colour and the differences in colour compared to both controls, baker’s flour and wholemeal flour, the Δ*E* value was calculated using the equation:

$$\Delta E=\sqrt{{\left(L_{2}^{*}-L_{1}^{*}\right)}^{2}+{\left(a_{2}^{*}-a_{1}^{*}\right)}^{2}+{\left(b_{2}^{*}-b_{1}^{*}\right)}^{2}\;}$$

where: 

L*_2_ = lightness value of the bread including EverVita ingredients; L*_1_ = lightness value of the control (baker’s flour or wholemeal flour).

a*_2_ = redness value of the bread including EverVita ingredients; a*_1_ = redness value of the control (baker’s flour or wholemeal flour).

b*_2_ = yellowness value of the bread including EverVita ingredients; b*_1_ = yellowness value of the control (baker’s flour or wholemeal flour).

#### 2.6.5. Water Activity and Microbial Shelf Life

The water activity was determined using a water activity metre (HygroLab, Rotronic, Bassersdorf, Switzerland). The microbial shelf life analysis of the breads were performed using the environmental challenge method as reported by Dal Bello et al. (2007) [28]. Briefly, breads were cut into 25 mm slices. Bread slices were exposed to the environment for 5 min on each side, followed by packing them separately in sterile plastic bag and heat sealing them. To ensure aerobic conditions and allow gas exchange, two filter pipettes were inserted in each bag. Mould growth was monitored for 14 days and visually evaluated, while samples were stored in a temperature-controlled room (20 ± 2 °C). Samples were rated as “mould-free”, “<10% mouldy”, “10–24% mouldy”, “25–49% mouldy”, “>50% mouldy”.

### 2.7. Impact of the Addition of EverVita Ingredients on the Nutritional Value of Breads

#### 2.7.1. Starch Composition, Predicted Protein and Fibre Content and Predicted Amino Acid Profile in Breads

Starch can be present in different forms, digestible and resistant towards digestion. The digestible starch and resistant starch content of freeze-dried bread samples were determined by an enzymatic method using the K-RAPRS kit (Megazyme, Bray, Ireland). The digestible and resistance starch content in fresh bread was calculated considering the moisture content. The sum of both starch components represents the total starch content. The predicted protein and fibre content were calculated based on nutritional information of the ingredients considering the bake loss. The expected indispensable amino acid (histidine, isoleucine, leucine, lysine, sulphur-containing amino acids (SAA), aromatic amino acids (AAA), threonine, tryptophan, valine) in the breads were calculated and are presented relative to the requirement pattern bread on an average intake of 0.66 g protein per kg composition in breads [29]. Therefore, the ratio of baker’s flour and EVF/EVP as well as the amino acid profile of the single ingredients were taken into account as reported by Hoehnel et al. (2020) [24]. 

#### 2.7.2. Predicted Glycaemic Index and Load

An in vitro digestion method, specific to fibre-enriched food, was carried out to determine the predicted glycaemic index (pGI) of the breads. The procedure was performed as described by Brennan and Tudorica (2008) [30] and 4 g of fresh bread crumb was used for analysis. The release of reducing sugars over time was measured spectrophotometrically and the pGI and the predicted glycaemic load (pGL) were calculated using the following formula:

(1) $$\begin{array}{ll} \mathrm{GI}=105.52 & \times \frac{\mathrm{fibre}}{\mathrm{digestible}\;\mathrm{carbohydrates}}-76.46\times \frac{\mathrm{protein}}{\mathrm{digestible}\;\mathrm{carbohydrates}}\\ & +1.23\times {\mathrm{RSRI}}_{\mathrm{at}\;150\;\min}+69.41\times {\mathrm{SDI}}_{\mathrm{at}\;270\;\min}-83.87 \end{array}$$

Reducing sugars released (RSR) is calculated as:

(2) $$\mathrm{RSR}\left[\%\right]=\frac{A_{\mathrm{sample}}*500*0.95}{A_{\mathrm{maltose}}*\mathrm{carbohydrates}}$$

where A_sample_ refers to the absorbance at 546 nm of the sample treated with enzymes; 500 refers to the total volume of buffer; 0.95 is the conversion factor of starch to maltose by amylase; A_maltose_ refers to the absorbance of a 1 mg/mL maltose standard; digestible carbohydrates in 4 g sample expressed in mg.

The reducing sugar release index (RSRI) at 150 min is defined as:

(3) $$\mathrm{RSRI}=\frac{\mathrm{RSR}\;150\min\;\left(\mathrm{Sample}\right)}{\mathrm{RSR}\;150\min\;\left(\mathrm{Control}\right)}\times 100$$

The ‘control’ refers to the baker’s flour control.

Sugar diffusion index:

(4) $$\mathrm{SDI}=\frac{{\mathrm{DIFF}}_{\mathrm{maltose}}}{{\mathrm{DIFF}}_{\mathrm{sample}+\mathrm{maltose}}}$$

where DIFF_maltose_ is the diffusion of the maltose blank (1 g maltose in 50 mL buffer). The percentage of maltose able to diffuse through the tube in the presence of sample (DIFF):

(5) $$\mathrm{DIFF}=\frac{\left(A_{\mathrm{blank}+\mathrm{maltose}}-{\;A}_{\mathrm{blank}}\right)\times 500}{A_{\mathrm{maltose}}\times 200}\times 100$$

where A_blank +maltose_ refers to the absorbance of the sample blank with maltose addition; A_blank_ refers to the absorbance of the sample blank; 500 is the total volume of buffer; A_maltose_ refers to the absorbance of the maltose blank; 200 is the weight of maltose in ‘blank + maltose’-sample in mg.

### 2.8. Statistical Evaluation

All tests were carried out in triplicate, unless stated otherwise. A variance analysis (one-way ANOVA, α ≤ 0.05, Tukey test) was performed using Minitab 17. In addition, a two-way ANOVA was conducted to evaluate the influence of the type of ingredients and the addition level. Correlation analysis was performed using Microsoft Excel.

## 3. Results

### 3.1. Nutritional Composition of Raw Ingredients

**Figure 1:**
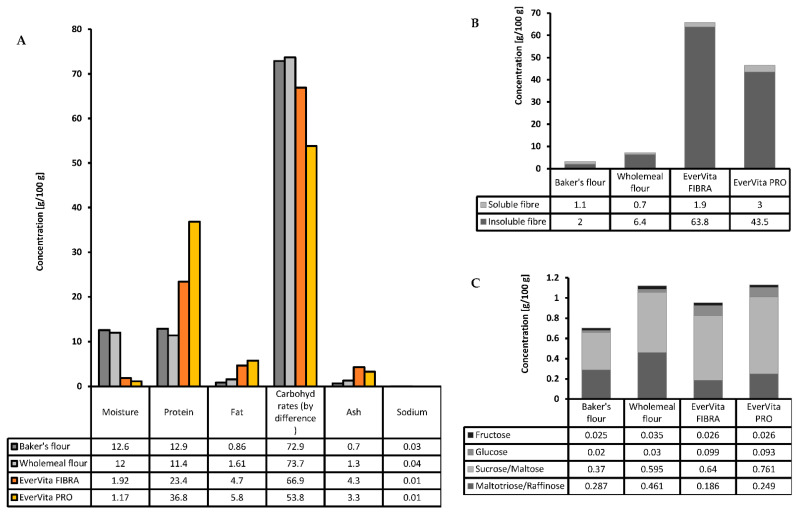
Composition of the raw ingredients baker’s flour, wholemeal flour and EverVita ingredients, EverVita FIBRA and EverVita PRO (**A**), including the sugar profile (**B**) and the amount of soluble and insoluble dietary fibre (**C**). Values are expressed as mean values with a coefficient of variation < 0.1.

The nutritional composition of the raw ingredients is an influencing factor of the final nutritional profile of the food product. The differences between baker’s flour (C1), wholemeal flour (C2) and EverVita ingredients regarding moisture, protein, fat, total carbohydrates, ash and sodium are illustrated in Figure 1A. EverVita ingredients are low in moisture (<2%) due to the processing (drying) of their original material BSG. Moreover, EverVita ingredients are exceptionally high in protein and fibre. EVF is by 81% and 105% higher in protein compared to C1 and C2, respectively. EVP contains an even greater amount, which is 185% and 222% of the protein content of C1 and C2, respectively. Both ingredients are similar in fat content (EVF: 4.7 g/100 g; EVP 5.8 g/100 g) and ash (EVF: 4.3 g/100; EVP: 3.3 g/100 g) which are higher compared to C1 and C2. The sodium level in EVF and EVP was lower than in C1 and C2. 

The total carbohydrate concentration in the EverVita ingredients is 6–19% lower compared to the flours, yet their dietary fibre content, illustrated in Figure 1B, is significantly higher. EVF has a total dietary fibre content of 65.7 g/100 g, which is by 21-fold and 9-fold higher than C1 and C2, respectively. 1.9 g/100 g of the total dietary fibre in EVF is soluble fibre, a concentration comparable with baker’s flour. EVP on the other hand contains less dietary fibre (46.5 g/100 g) than EVF, which is still significantly higher compared to C1 (3.3 g/100 g) and C2 (7.1 g/100 g). The soluble fibre content in EVP is 3 g/100 g, which is 2.7-fold and 4.2-fold the amount present in C1 and C2, respectively. Besides dietary fibre, mono-, di- and tri-saccharides are present in the raw ingredients (Figure 1C). Baker’s flour showed the lowest total sugar content (0.702 g/100 g), followed by EVF (0.951 g/100 g). Wholemeal flour and EVP showed the highest sugar contents (1.12 g/100 g and 1.13 g/100 g, respectively). Both EverVita ingredients had a higher content of sucrose/maltose compared to C1 and C2.

**Figure 2:**
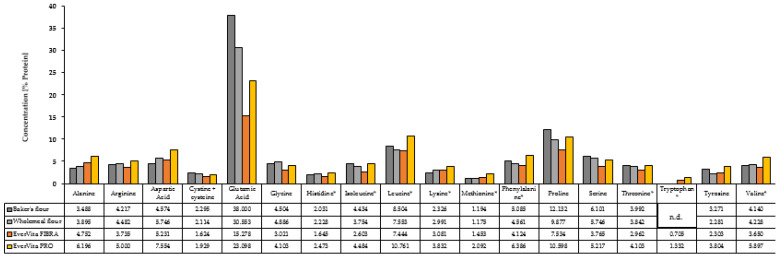
Amino acid composition of the raw ingredients baker’s flour, wholemeal flour and EverVita ingredients, EverVita FIBRA and EverVita PRO, in % based on protein. Values are expressed as mean values with a coefficient of variation < 0.1. * Indispensable amino acids. n.d. stands for ‘not detected’.

Apart from the macronutrients, such as protein, fat, and carbohydrates, the protein profile plays a key role in the final nutritional value of food products. The total amino acid composition of the protein fraction after hydrolysis of the raw ingredients based on their protein content is displayed in Figure 2. Fourteen of 18 amino acids showed their highest content in EVP among all raw ingredients. The concentrations were 1–75% higher compared to C1 and C2. A special emphasis needs to be put on the amount of indispensable amino acids which were all present in a higher amount in EVP compared to the other ingredients. In particular leucine and phenylalanine which make up 10.76% and 6.39% of the protein of EVP. Furthermore, both, EVP and EVF, contain tryptophan which was not detected in baker’s flour or wholemeal flour. 

### 3.2. Impact of EverVita Ingredients on Gluten Network Formation and Starch Pasting

**Figure 3:**
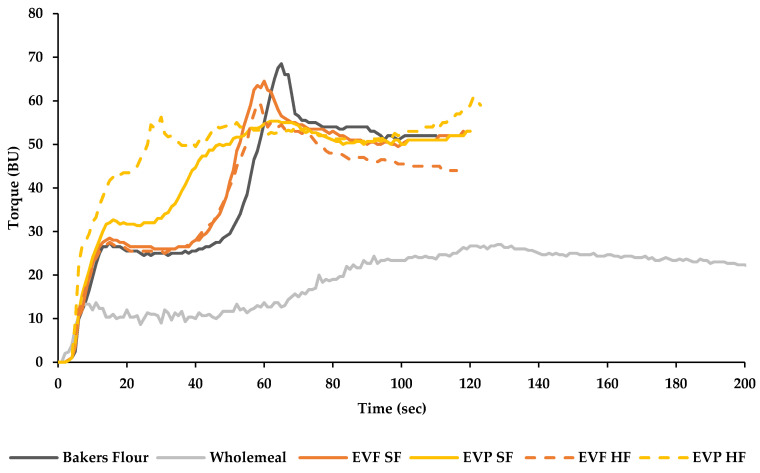
Gluten network development of baker’s flour formulation enriched with EverVita ingredients (EverVita FIBRA (EVF), EverVita PRO (EVP)) at two inclusion levels (source of fibre (SF), high in fibre (HF)) compared to baker’s flour control and wholemeal flour control. The curves represent average torque values of triplicates per sample. Fibre fortification using EverVita ingredients influenced the starch pasting properties as demonstrated in Table 2. Chosen decisive parameters are peak viscosity, breakdown viscosity, trough viscosity and final viscosity. The peak viscosity represents the maximum viscosity during heating and shearing and refers to the water binding capacity and swelling power of the starch. C1 showed the highest peak viscosity (1209 ± 9 cP) among the samples, while C2 showed the lowest (599 ± 33 cP). The incorporation of EverVita ingredients led to a significant lower degree of swelling compared to C1. This effect was advanced by HF addition levels of EVF (865 ± 2 cP) and EVP (812 ± 4 cP). After the peak viscosity, amylose and amylopectin leach out of the starch granules causing a decrease in viscosity to a certain trough. The trough viscosity indicates the holding strength of the system before retrogradation occurs.

The effect of EverVita ingredients on gluten network development compared to the controls baker’s flour (C1) and wholemeal flour (C2) is illustrated in Figure 3. C1 showed a gluten network development as commonly seen in refined wheat flour [31,32,33], characterised by an immediate increase in torque (flour hydration), followed by a plateau (colliding of gliadins and glutenins), and a further increase reaching a torque maximum (TM) at 68.00 ± 0.00 BU after 65.00 ± 0.00 s (PMT). C2 resulted in a different curve pattern with a significantly lower TM (27.67 ± 1.15 BU) and a significantly higher PMT (126.00 ± 7.55 s) compared to all other samples. The replacement of baker’s flour by EverVita ingredients caused changes in gluten network development, particularly when EVP was used. Changes intensified with increasing addition level of the ingredients. The curve pattern of EVF inclusion appeared to be similar to C1 reaching lower TM-values of 65.5 ± 0.7 BU and 52.2 ± 2.1 BU, in SF and HF formulations respectively. EVF addition led to faster gluten network development with 60.5 ± 2.1 s in EVF (SF) and 60.5 ± 0.7 s in EVF (HF). The incorporation of EVP resulted in a significantly lower TM compared to C1, but addition level did not impact the result (55.7 ± 0.6 BU (SF); 55.7 ± 1.2 BU (HF), while the concentration significantly (*p* < 0.05) influenced the PMT (63.3 ± 2.3 s (SF); 29.0 ± 1.0 s (HF)).

C1 showed the highest trough viscosity (740 ± 6 cP), followed by formulations including EVF (SF) (655 ± 2 cP) and EVP (SF) (645 ± 1 cP). The incorporation of EVP (HF) showed a significant lower trough viscosity (465 ± 4 cP). The trough viscosity of C2 (516 ± 15 cP) was lower compared to C1 and did not significantly differ from samples including EVF (HF) (530 ± 10 cP). During cooling amylose and amylopectin reassociate, which is called retrogradation, and leads to an increase in viscosity. The highest final viscosity was determined in the C1 (1758 ± 9 cP), followed by the formulations with EverVita ingredients at a SF level (EVF (SF): 1603 ± 8 cP; EVP (SF): 1606 ± 1 cP). The addition of EVF at a higher level (HF) led to a significant lower value (1315 ± 8 cP) which is comparable to the final viscosity detected in C2 (1318 ± 28 cP). The incorporation of EVP at HF level resulted in the lowest final viscosity (1222 ± 11 cP).

### 3.3. Rheological Properties of Doughs and Fermentation Quality

Rheological characteristics of dough include consistency changes during mixing (Farinograph) as well as elasticity and resistance to extension during stretching of the dough (Extensograph).

**Figure 4:**
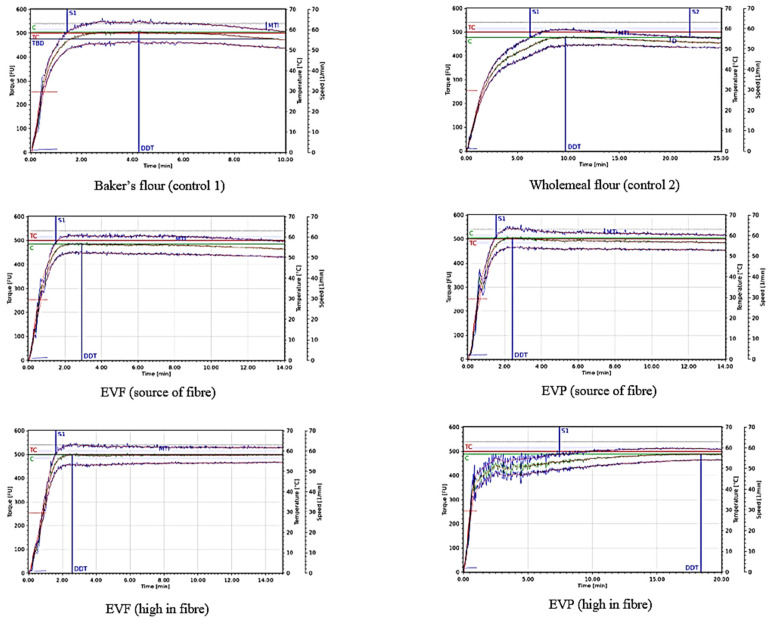
Farinograph curves of the controls (baker’s flour and wholemeal flour) and the impact of EverVita FIBRA (EVF) and EverVita PRO (EVP) on dough rheology during mixing.

The farinograph was not only used to adjust the water content (WA) of each dough reaching a final consistency of 500 ± 20 FU, rheological parameters such as dough development time (DDT), arrival of dough stability (S1) and the mixing tolerance index (MTI) were investigated. Figure 4 shows the Farinograph curves of the different formulations and the results of DDT, S1 and MTI are shown in Table 2. 

C1 showed the lowest water absorption resulting in a water addition level of 60.47% to achieve 500 FU, while C2 required 63.30% water. The addition of EVF (SF) and EVP (SF) caused an increased water addition level by 1.80% and 3.50% compared to C1, respectively. While the incorporation of EVF (HF) only affected the water absorption of the system to a relatively small extent (+3.83%) compared to C1, EVP (HF) caused an increase by 10.26% and showed the highest water content overall. DDT and S1 revealed a strong positive correlation (*p* < 0.04; *r* = 0.95). C2 resulted in a longer DDT (10.12 ± 0.28 min) and a delay in S1 (6.93 ± 0.32 min) compared to C1 (DDT: 4.57 ± 0.28; S1: 1.42 ± 0.13 min). Both EverVita ingredients caused a quicker DDT (EVF: 2.51 ± 0.37 min; EVP: 2.48 ± 0.09 min) and S1 is reached earlier when added in ‘source of fibre’ levels (EVF (SF): 1.33 ± 0.12 min; EVP (SF): 1.27 ± 0.12 min). While the addition level of EVF did not have any impact on the dough rheology during mixing, the longest overall DDT (17.84 ± 0.65 min) and the highest S1 (8.07 ± 0.98 min) occurred in doughs with EVP (HF). The MTI showed the highest value for C1 (34.33 ± 1.53) while all other doughs had a significantly lower MTI (between 13.00 to 19.67).

The Farinograph measurement of EVP (HF) did not give any MTI value which indicates no changes in peak consistency five minutes after the peak is reached. Furthermore, the curve showed fluctuations in the first 7.5 min of mixing.

Extensograph measurements revealed the extensibility (E) and the resistance to extension (RE) during stretching of the dough and the results are demonstrated in Table 2. C1 showed the highest extensibility (167.00 ± 2.94 EU), followed by EVF (SF) (144.25 ± 6.90 EU) and EVP (SF) (131.75 ± 7.23 EU). The lowest extensibility was detected in C2 (63.00 ± 2.94 EU) and EVP (HF) (79.00 ± 2.45 EU), while EVF (HF) resulted in a significantly higher dough extensibility (117.25 ± 2.06 EU). The RE value in C1 was the lowest (168.00 ± 9.63 mm), while EVP (HF) showed the highest RE (840.00 ± 36.00 mm). The ratio of RE over extensibility indicates the balance between dough strength and dough stretchability. The ratio in C1 was 1.01 ± 0.07. The addition of EverVita ingredients, especially EVP, increased the value resulting in the highest RE/E in EVP (HF) (10.64 ± 0.57 mm/EU).

The rheofermentometer was used to measure dough rise and CO2 formation during 180 min of fermentation. The results for maximum height of the dough (Hm), time required to achieve maximum height (T1), the total volume of carbon dioxide released by the dough (Vtot) and the height of maximum gas formation (H’m) are displayed in Table 2. C1 showed the greatest dough height during the leavening process (Hm = 77.50 ± 1.05 mm), while dough rise of C2 resulted in the lowest Hm value (16.63 ± 1.96 mm). The addition of EverVita ingredients caused a significantly lower Hm, especially when EVP was applied. This effect was amplified by a higher addition level of the ingredients leading to values of 53.70 ± 2.57 mm and 25.6 ± 1.4 mm in EVF (HF and EVP (HF), respectively. The time at which the maximum dough height was achieved was 88.50 ± 6.36 min in C1 which did not differ significantly from C2 (71.00 ± 3.12 min). The maximum gas formation did not differ significantly in the doughs except for EVP (HF) which showed a significantly lower H’m (126.9 ± 6.3 mm) compared to all other samples. This resulted in the same trend for Vtot with C1 (2601 ± 40 mL) and EVP (SF) (2585 ± 85 mL) showing the highest CO_2_ production by yeast, while in dough including EVP (HF) the lowest CO_2_ formation occurred (2426 ± 48 mL).

### 3.4. Effect of EverVita FIBRA and EverVita PRO on Bread Quality

The specific volume, crumb texture, crumb macro- and microstructure as well as the crust and crumb colour, water activity and microbial shelf life of the breads were determined to evaluate bread quality. The results are illustrated in Table 3.

**Figure 5:**
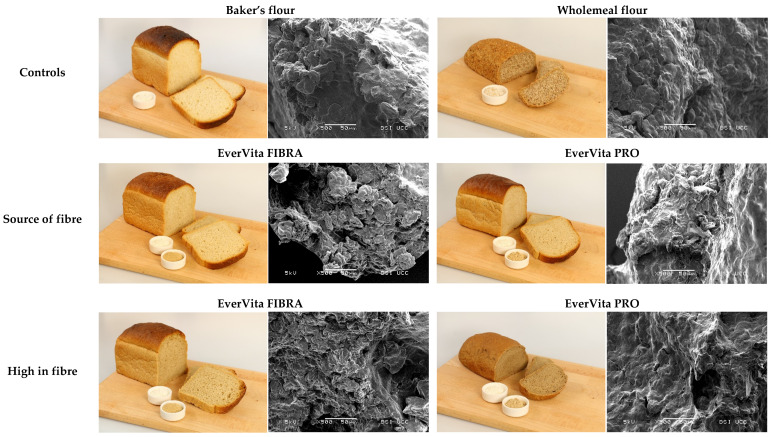
Appearance of fresh bread samples and micrographs of the bread crumbs. The first row illustrates the controls, baker’s flour and wholemeal flour; Breads including EverVita FIBRA and EverVita PRO in source of fibre level and in high in fibre level are demonstrated in the second and third row, respectively.

The addition of EverVita ingredients in a concentration to achieve a ‘source of fibre’ claim did not significantly impact the specific volume (EVF (SF): 4.66 ± 0.23 mL/g; EVP (SF): 4.38 ± 0.31 mL/g) compared to C1 (4.46 ± 0.26 mL/g). However, a higher addition level decreased the specific volume of the breads significantly resulting in 3.72 ± 0.37 mL/g and 2.17 ± 0.05 mL/g for EVF (HF) and EVP (HF) breads, respectively. EVP (HF) showed the same specific volume as C2 (2.28 ± 0.07 mL/g). In Figure 5 the differences in volume are visualised.

The texture of the bread crumb was evaluated considering crumb hardness (N) and chewiness (N), and the degree of staling over time was determined and expressed as the staling rate. The softest crumb was determined in C1 (4.76 ± 1.20 N) and EVF (SF) (4.77 ± 0.65 N), followed by EVP (SF) (6.25 ± 1.49 N) and EVF (HF) (9.03 ± 1.23 N). C2 showed a significant harder crumb (24.54 ± 3.68 N) and the highest crumb hardness was measured in bread containing EVP (HF) (36.36 ± 1.99 N). EVF (SF) resulted in the least chewy crumb (3.25 ± 0.56 N), while EVP (HF) had the highest chewiness value (20.32 ± 1.20 N). The staling rate was the highest in EVF (SF), EVP (SF) and EVF (HF) with values between 2.55 and 2.70. The lowest staling rates were detected in C2 (1.32 ± 0.45) and EVP (HF) (1.24 ± 0.15). 

Changes in crumb structure were evaluated considering slice area (mm^2^), number of cells and average cell diameter (mm). A strong positive correlation between slice area and specific volume (*p* < 0.003; *r* = 0.99) occurred and thus the biggest slice area was measured in C1 (10,323 ± 590 mm^2^) and EVF (SF) (10,469 ± 432 mm^2^) breads, while the smallest slice area was determined in C2 (4990 ± 388 mm^2^) and EVP (HF) (5701 ± 369 mm^2^). The highest number of cells in the bread crumb was determined in EVP (SF) (5997 ± 268). These cells were relatively small (diameter of 1.88 ± 0.10 mm) compared to those of the other samples. C2 showed the lowest number of cells (2794 ± 144) and also the smallest average cell diameter (1.25 ± 0.20 mm). C1 and EVF (SF) had the biggest cell diameter among all samples with 2.43 ± 0.20 mm and 2.32 ± 0.12 mm for C1 and EVF (SF), respectively. Differences in crumb structure were visualised using a SEM and are illustrated in Figure 5. The inclusion of EVF resulted in a crumb structure very similar to C1, while EVP caused a compact and dense crumb structure with a film covering the starch granules. Furthermore, the crumb surface occurred to be bigger overall in breads with EVF compared to EVP, in particular with high in fibre inclusion levels. 

The crust and crumb colour of the breads were evaluated by determining the ΔE-value which indicated the difference in L*-, a*- and b*-value compared to C1 and C2 as controls. Compared to C1, C2 showed the biggest difference in crust colour (11.17 ± 2.65) and crumb colour (17.66 ± 2.86). The most similar crust colour to C1 was determined in breads including EVP (HF) (8.21 ± 4.47), while EVF (SF) (5.28 ± 3.39) and EVP (SF) (8.92 ± 2.80) showed the most similar crumb colour to C1. Compared to C2, breads containing EverVita ingredients with source of fibre addition level (EVF (SF): 15.08 ± 2.42; EVP (SF): 16.54 ± 2.55) had the highest ΔE-value, while EVP (HF) showed the most similar crust colour (8.93 ± 2.30). Regarding the colour difference in crumbs, EVP (HF) had the most similar crumb colour to C2 (5.69 ± 2.20), whereas C1 showed the highest difference. 

The microbial shelf life of the breads revealed the shortest shelf life in C1 and EVF (SF) breads. Compared to C1 which showed the first microbial growth after 6.00 ± 1.00 days, the growth started after 9.00 ± 1.00 days and 9.33 ± 0.58 days in breads including EVP (SF) and EVP (HF), respectively. Hence, an extension of microbial shelf life occurred, even though no significant differences in water activity was determined.

### 3.5. Modification of Nutritional Value of Breads

The nutritional value of breads was evaluated considering the composition of the breads (starch composition, protein and fat content, moisture content) as well as the amino acids composition and the predicted glycaemic index (pGI) and predicted glycaemic load (pGL).

The predicted composition of the breads based on ingredient characteristics and the addition level of EverVita ingredients is illustrated in Table 4.

The main compound of the breads is moisture, which ranges between 42.04 ± 0.04% in EVF (SF) breads to 46.68 ± 0.26% in EVP (HF) breads. The second main compound in the breads is starch. The highest total starch content was determined in C1 (40.56 ± 0.42 g/100 g), whereas high fibre breads and C2 showed the lowest total starch concentrations. The same trend occurred in the digestible starch content. C1 had the lowest dietary fibre content (2.1 g/100 g) and C2 included 4.8 g/100 g dietary fibre based on calculation. EverVita ingredients were added in amounts needed to achieve either 3 g/100 g of dietary fibre (‘source of fibre’ claim) or 6 g/100 g (‘high in fibre’ claim). The inclusion of EverVita ingredients increased the protein content in the breads, which ranged between 8.4 g/100 g (C2) and 11.5 g/100 g (EVP (HF)). No major differences occurred in the fat content of the breads. C1 had the lowest fat content (3.18 g/100 g) and EVP (HF) breads included the highest fat content (3.47 g/100 g).

**Figure 6:**
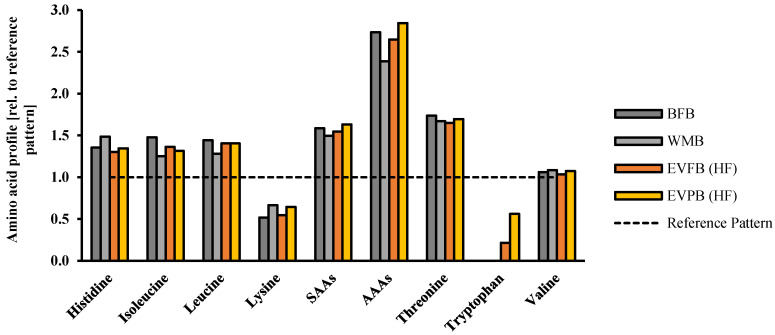
Profile of indispensable amino acids of the final breads baker’s flour control (BFB), wholemeal flour control (WMB), high in fibre bread including EverVita FIBRA (EVFB (HF)) and high in fibre bread with EverVita PRO addition (EVPB (HF)). The values are expressed relative to the requirement pattern established by the World Health Organisation [29].

The amount of indispensable amino acids was calculated and expressed relative to the requirement pattern established by the World Health Organisation (WHO) (Figure 6). Since the inclusion level of ‘source of fibre’ breads including EverVita ingredients are relatively low, changes in amino acid profile were expected rather for ‘high in fibre’ breads; hence, source of fibre breads were neglected in the evaluation of the predicted amino acid composition of the final breads. The replacement of baker’s flour by any of the EverVita ingredients did not result in an inferior amino acid score. 

By contrast, the fortification of wheat bread with EVP increased the concentration of some indispensable amino acids, in particular lysine (+24.5%) an amino acid that is known to be limiting in cereal based products. Moreover, the incorporation of EVP increased the concentration of aromatic amino acids (AAA) by 4% compared to C1, and valine (+1%). A significant difference occurred in the predicted tryptophan content in both high in fibre breads (EVF and EVP), which made 0.215% and 0.561% based on the total protein content in the breads, respectively, while no tryptophan was expected in both controls.

**Figure 7:**
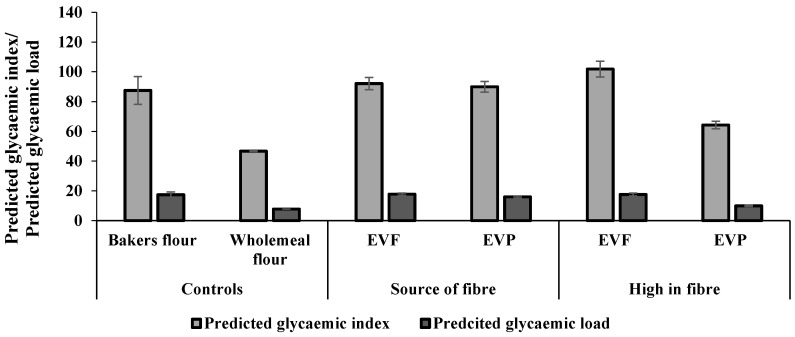
The predicted glycaemic index/glycaemic load of bread samples. EVF refers to EverVita FIBRA, while EVP represents EverVita PRO. SF and HF indicate the addition level of EverVita ingredients at ‘source of fibre’ and ‘high in fibre’, respectively.

The pGI and pGL values represent the in vitro starch digestibility of the breads during digestion. The pGI and pGL of the different bread samples are illustrated in Figure 7. C1 showed a pGI value of 87.49 ± 9.31, which was significantly higher than C2 with a pGI of 46.75 ± 0.20. The replacement of baker’s flour by EverVita ingredients aiming for a ‘source of fibre’ claim according to EU regulations did not result in an inferior bread quality regarding starch digestibility. On the opposite: EVP (HF) breads caused a significant reduction in pGI (64.25 ± 2.53). The same trend was observed for the pGL values, with C2 (7.75 ± 0.03) and EVP (HF) (9.97 ± 0.39), which can be considered as low. 

## 4. Discussion

The impact of BSG on bread dough characteristics and final bread quality has been extensively studied. Rejuvenated BSG in the form of two ingredients, EverVita FIBRA and EverVita PRO, was used to fortify bread in two inclusion levels, 3 g/100 g and 6 g/100 g bread. The type of EverVita ingredient significantly impacted on dough and bread quality and final bread characteristics.

EVF contains 23.4% protein and 67.6% dietary fibre, mainly insoluble fibre, while EVP has a protein content of 36.8% and a dietary fibre content of 46.8% [23]. Both ingredients weakened the gluten network. The inclusion level was not the only parameter impacting network formation (*p* < 0.001), the type of EverVita ingredient also influenced the network strength (*p* < 0.005) and development time (*p* < 0.001). As previously reported, the EverVita ingredients contain low molecular weight peptides, which promote intramolecular connections, such as disulphide-, hydrogen- and ionic bonds resulting in a faster network development [23,34]. Since EVP has a higher protein content compared to EVF, it shortened the development time more effectively. EVF, on the other hand, influenced the network strength somewhat. Compared to EVP, EVF is higher in dietary fibre, that is mainly insoluble, which causes firstly changes in the secondary structure of gluten proteins due to the formation of hydrogen bonds between β-turns and the fibre, causing a weaker network [35,36]. Secondly, fibre competes with gluten for water leading to a lower degree of gluten hydration and hence a restricted network development [37]. Also, a physical hindrance of the gluten network development may occur, which was detected to a higher extent in wholemeal flour (C2) due to the presence of bran particles [38,39]. 

The gluten network strength influenced the dough rheology resulting in a positive correlation with extensibility (*p* < 0.036; *r* = 0.84) and dough rise during proving (*p* < 0.039; *r* = 0.84). This indicates the primary role of the gluten network on dough quality and the weakening effect caused by fibre ingredients [40,41,42,43]. Bread dough is considered ideal in its viscoelastic properties if the ratio of viscous and elastic parts is balanced, meaning RE/E is 1 [44]. Compared to EVF, EVP increased the resistance to extension enormously. Thus, the dough’s elastic part increased, causing a higher dough stiffness, most likely due to the higher protein content. Proteins can form covalent bonds with other proteins and peptides [45] which increases the resistance of the system to external stress [46,47], such as mixing, for example, which is illustrated by the mixing tolerance index. This index indicates that formulations including EVP withstand the mixing to a greater extent than all other formulations. 

The dough rise during fermentation (Hm) is influenced by both, addition level (*p* < 0.0001) and the type of EverVita ingredient (*p* < 0.0001) and showed a strong positive correlation with the gluten network strength (*p* < 0.038; *r* = 0.84). Baker’s flour is rich in intact gluten proteins and starch, which participate in dough structure formation [48]. The replacement of flour by EVP or EVF caused a reduction of both intact gluten and starch in the dough system and reduced its viscoelastic behaviour. The replacement by EVF increased the amount of insoluble fibre in the system interacting with the gluten network. This weakened the network mainly due to physical interaction and lowered DDT [49,50]. On the other hand, the substitution by EVP caused a lower Hm due to an increase in resistance to extension of the dough proven by a strong positive correlation with RE/E (*p* < 0.04; *r* = −0.84). Furthermore, the curves of the gluten network development (Figure 3) illustrate a very high resistance of formulations including EVP reflected by no sudden breakdown of the network, which was also found in bread systems fortified with plant-based proteins [31]. Moreover, the smaller particle size allowed a higher degree of interaction with compounds, such as water, fibre, and other proteins [51]. It also needs to be mentioned that the addition level of EVP to reach the fibre claims was higher compared to EVF, which led to a reduction of wheat flour and related gluten concentration in the final system. This reduction also influenced the colour formation during baking which was mainly affected by the colour of the ingredients EVF and EVP.

Vtot is the total volume of CO_2_ produced by yeast during proving, mainly influenced by the amount of available carbohydrates as a nutritional substrate for yeast. The replacement of baker’s flour, which contains over 70% carbohydrates, mainly starch, by ingredients containing less than 5% starch [23], resulted in less available carbohydrates to be metabolised by yeast, and hence less CO_2_ production. Thus, Vtot is only affected by the flour replacement level by EverVita ingredients (*p* < 0.01).

It is known that dough extensibility and dough expansion during proofing influence the final bread quality. Hm and RE/E correlated with several bread quality parameters, such as specific volume (*p* < 0.003; *r* = 0.96), crumb hardness (*p* < 0.018; *r* = −0.89), chewiness (*p* < 0.05; *r* = −0.83) and slice area (*p* < 0.0005; *r* = 0.98). The specific volume correlated negatively with the dough development time during mixing (*p* < 0.02; *r* = −0.88). To achieve the highest specific volume the formulation can reach, the dough needs to be fully developed, meaning all compounds need to be fully hydrated to form a network [52]. EVP (HF) showed a significantly long DDT, putatively due to the incorporation of high amounts of low molecular weight proteins competing with other compounds for water and hence delaying the hydration of the system [53]. This indicates that the mixing time used to prepare the bread dough might have been insufficient for all formulations resulting in a low specific volume [54]. EVP caused a significant reduction in specific volume, which led to a denser crumb structure, resulting in a harder crumb and a higher crumb chewiness, which both correlated negatively with the specific volume (*p* < 0.01; *r* = 0.92).

Besides the dough rheology, starch pasting during baking and the related changes in viscosity of the system impacts the final product quality. In general, the replacement of baker’s flour caused lower viscosity values which may be related to the reduced amount of total starch. The peak viscosity showed a strong positive correlation with the specific volume of the bread (*p* < 0.02; *r* = 0.89), meaning a high degree of starch swelling during heating results in a high viscosity which enhances the stability of the system during the baking process [55], and leads to a high specific volume. The incorporation of EVP resulted in a lower peak viscosity due to the higher protein content and the related higher water absorption than EVF, causing an increasing competition with starch for water and a restricted starch swelling [56]. 

As already mentioned, EVP (HF) caused a significant decrease in the specific volume of the breads, which affected the crumb structure. Food structure affects the starch digestibility of products making starch less accessible for digestive enzymes to bind on the substrate, in this case, starch [57]. In particular, proteins are known to form a matrix in which starch granules are embedded. This appears to be a barrier towards enzymatic starch degradation [57]. Also, during the baking process, changes in protein conformation may occur, which may promote the formation of disulphide bonds [58]. Thus, EVP showed a high impact on reducing the pGI-value and the pGL. The partial replacement of baker’s flour by EVF did not decrease the pGI, but it also did not result in an inferior bread quality regarding starch digestibility compared to C1. EVF not having any impact on the pGI could have been firstly, by a lower protein content and thus a weaker protein matrix protecting the starch granules from enzymatic attack [59], and secondly, by the bigger particle size of the ingredients causing a higher degree of physical interruption of the protein-starch network [60]. Micrographs of the crumb (Figure 5) also demonstrate the higher overall surface in bread with EVF compared to EVP, which increases the chance of enzymatic degradation. EVP not only influenced the starch digestibility positively, the addition of EVP in ‘high in fibre’ level also elevated the amount of indispensable amino acids in the bread. This was especially pronounced for lysine and tryptophan, which are known to be limited in cereal-based products [61]. Tryptophan is present in barley in higher amounts compared to wheat [62]. Hence, by replacing baker’s flour with BSG-derived ingredients, such as EVF and EVP, a natural tryptophan fortification occurs. This indispensable amino acid contributes to protein synthesis in the human body and is involved in other physiological mechanisms, such as the synthesis of serotonin and vitamin B_3_ [62,63]. In addition, dietary tryptophan has a high therapeutic potential to treat multiple chronic diseases such as cardiovascular disease, depression and inflammatory bowel disease, among others [62]. Even though the requirements for those two indispensable amino acids proposed by the WHO are not reached, the amino acid score increased by the replacement of baker’s flour by EVP. Hence, a high-fibre bread with elevated protein quality was achieved.

In addition, the structural differences could explain the differences in microbial shelf life. The protein matrix, putatively, also acts as a barrier for microbial attack on starch, prolonging the shelf life by two to three days. Apart from the structural influence, the increased water binding capacity of fibre and especially proteins restrict the accessibility to water and hinder the growth of mould [64]. 

## 5. Conclusions

BSG is a nutritious side-stream of the brewing industry that is used primarily for animal feed. Previously, using BSG as a food ingredient to enrich products, particularly cereal products, with highly nutritious dietary fibre and protein has been very challenging, more often than not leading to a significant decrease in final bread quality. The present study used two unique BSG-derived ingredients, EverVita FIBRA (EVF) and EverVita PRO (EVP), which differ in protein and dietary fibre content as well as particle size. These two ingredients performed very similarly in small inclusion levels but differently in bread systems with a ‘high in fibre’ addition level. The inclusion of EVF in high amounts resulted in outstanding bread characteristics such as high specific volume, soft crumb texture and a crumb structure comparable to a baker’s flour control. While the impact of EVP on the techno-functional properties was less desired, it significantly increased the nutritional value of the bread by increasing protein content by 36% and fibre content by 3-fold, lowering pGI-values by up to 25%, elevating the amount of indispensable amino acids and hence the protein quality, and prolonging microbial shelf life. Notwithstanding this, correlation analysis revealed the influence of dough characteristics on bread quality which can help make controlled changes in the baking process or even the formulation for further optimisation using functional ingredients. In the past, several studies have addressed the impact of the most abundant dietary fibre in BSG, arabinoxylans, on bread quality and the interaction between fibre and gluten network. However, the impact of the protein fraction present in BSG has been neglected in the literature. This study provided a deep insight into the impact of several BSG-derived fractions, including protein, on bread quality. This work demonstrated the potential game-changing impact of brewing side-streams to sustainably enhance the nutritional value of food products.

## Figures and Tables

**Table 1:** Recipes of breads in % based on baker’s flour/wholemeal flour + EverVita ingredient.

	Controls		Source of Fibre		High in Fibre	
Ingredients	Baker’s Flour (C1)	Wholemeal Flour (C2)	EverVita FIBRA	EverVita PRO	EverVita FIBRA	EverVita PRO
Bakers flour	100.00	n.a.	96.00	95.00	89.00	84.00
Wholemeal flour	n.a.	100.00	n.a.	n.a.	n.a.	n.a.
EverVita ingredient	n.a.	n.a.	4.00	5.00	11.00	16.00
Sunflower oil	3.20	3.20	3.20	3.20	3.20	3.20
Salt	1.20	1.20	1.20	1.20	1.20	1.20
Sugar	2.00	2.00	2.00	2.00	2.00	2.00
Bakers yeast	2.00	2.00	2.00	2.00	2.00	2.00
Water (25 °C)	60.47	63.30	62.27	63.97	64.30	70.73

n.a. represents ‘not applicable’

**Table 2:** Pasting properties and dough analysis of samples. The results show mean values ± standard deviations. Values with the same lower-case letter in a row do not differ significantly from each other.

	Controls		Source of Fibre		High in Fibre	
	Bakers Flour (C1)	Wholemeal Flour (C2)	EverVita FIBRA	EverVita PRO	EverVita FIBRA	EverVita PRO
Peak Viscosity (cP)	1209 ± 9 (a)	599 ± 33 (e)	1077 ± 12 (b)	1067 ± 13 (b)	866 ± 2 (c)	812 ± 4 (d)
Breakdown (cP)	469 ± 4 (a)	101 ± 2 (d)	422 ± 10 (b)	422 ± 12 (b)	336 ± 8 (c)	347 ± 9 (c)
Trough (cP)	740 ± 6 (a)	516 ± 15 (c)	655 ± 2 (b)	645 ± 1 (b)	530 ± 10 (c)	465 ± 4 (d)
Final viscosity (cP)	1758 ± 9 (a)	1318 ± 28 (c)	1603 ± 8 (b)	1606 ± 1 (b)	1315 ± 8 (c)	1222 ± 11 (d)
Water absorption (%)	60.47 ± 0.15 (e)	63.30 ± 0.36 (c)	62.27 ± 0.25 (d)	63.97 ± 0.31 (bc)	64.30 ± 0.17 (b)	70.73 ± 0.25 (a)
Dough development time (min)	4.57 ± 0.28 (c)	10.12 ± 1.12 (b)	2.51 ± 0.37 (d)	2.48 ± 0.09 (d)	2.57 ± 0.02 (d)	17.84 ± 0.65 (a)
Dough stability at arrival (min)	1.42 ± 0.13 (b)	6.93 ± 0.32 (a)	1.33 ± 0.12 (b)	1.27 ± 0.12 (b)	1.50 ± 0.10 (b)	8.07 ± 0.98 (a)
Mixing tolerance index (FU)	34.33 ± 1.53 (a)	18.67 ± 6.35 (bc)	13.00 ± 2.83 (c)	19.67 ± 1.53 (b)	13.00 ± 4.00 (c)	Not detected
Extensibility (mm)	167.00 ± 2.94 (a)	63.00 ± 2.62 (f)	144.25 ± 6.90 (b)	131.75 ± 7.23 (c)	117.25 ± 2.06 (d)	79.00 ± 2.45 (e)
Resistance to extension (EU)	168.00 ± 9.63 (d)	363.25 ± 119.29 (bc)	260.00 ± 54.95 (cd)	350.25 ± 39.20 (bc)	425.50 ± 17.16 (b)	840.00 ± 36.00 (a)
Resistance to extension/Extensibility (EU/mm)	1.01 ± 0.07 (d)	5.75 ± 1.80 (b)	1.80 ± 0.33 (d)	2.66 ± 0.25 (cd)	3.63 ± 0.16 (c)	10.64 ± 0.57 (a)
Hm (mm)	77.50 ± 1.05 (a)	16.63 ± 1.96 (e)	68.80 ± 1.37 (b)	58.57 ± 2.50 (c)	53.70 ± 2.57 (c)	25.6 ± 1.4 (d)
T1 (min)	88.50 ± 6.36 (b)	71.00 ± 3.12 (b)	104.25 ± 3.18 (b)	172.50 ± 7.79 (a)	177.75 ± 3.18 (a)	175.5 ± 6.5 (a)
H’m (mm)	136.27 ± 3.22 (ab)	139.07 ± 2.51 (a)	138.47 ± 0.81 (ab)	139.07 ± 5.95 (a)	131.43 ± 3.76 (ab)	126.9 ± 6.3 (b)
Vtot (mL)	2601 ± 40 (a)	2498 ± 10 (ab)	2570 ± 46 (ab)	2585 ± 85 (a)	2476 ± 70 (ab)	2426 ± 48 (b)

**Table 3:** Technological properties of the bread samples. The results show mean values ± standard deviations. Values with the same lower-case letter in a row do not differ significantly from each other.

	Controls		Source of Fibre		High in Fibre	
	Bakers Flour (C1)	Wholemeal Flour (C2)	EverVita FIBRA	EverVita PRO	EverVita FIBRA	EverVita PRO
Specific Volume (mL/g)	4.46 ± 0.26 (a)	2.28 ± 0.07 (c)	4.66 ± 0.23 (a)	4.38 ± 0.31 (a)	3.72 ± 0.37 (b)	2.17 ± 0.05 (c)
Hardness (N)	4.76 ± 1.20 (e)	24.54 ± 3.68 (b)	4.77 ± 0.65 (e)	6.25 ± 1.49 (de)	9.03 ± 1.28 (cd)	36.36 ± 1.99 (a)
Chewiness (N)	8.39 ± 1.67 (c)	14.24 ± 1.79 (b)	3.25 ± 0.56 (f)	4.32 ± 1.26 (e)	6.40 ± 1.18 (d)	20.32 ± 1.20 (a)
Staling rate (-)	1.60 ± 0.31 (b)	1.32 ± 0.45 (bc)	2.66 ± 0.42 (a)	2.70 ± 0.51 (a)	2.55 ± 0.49 (a)	1.24 ± 0.15 (c)
Slice Area (mm^2^)	10323 ± 590 (a)	4990 ± 388 (e)	10469 ± 432 (a)	9677 ± 640 (b)	8970 ± 568 (c)	5701 ± 369 (d)
Number of Cells	5228 ± 349 (b)	2794 ± 144 (c)	5434 ± 383 (b)	5997 ± 268 (a)	5234 ± 296 (b)	5336 ± 514 (b)
Cell Diameter (mm)	2.43 ± 0.20 (a)	1.25 ± 0.20 (d)	2.32 ± 0.12 (a)	1.88 ± 0.10 (c)	2.08 ± 0.11 (b)	1.31 ± 0.13 (d)
ΔE crust (compared to C1)	-	11.17 ± 2.65 (a)	10.91 ± 2.11 (ab)	10.95 ± 1.87 (ab)	9.03 ± 1.93 (ab)	8.21 ± 4.47 (b)
ΔE crust (compared to C2)	11.17 ± 2.65 (b)	-	15.08 ± 2.42 (a)	16.54 ± 2.55 (a)	11.27 ± 1.46 (b)	8.93 ± 2.30 (b)
ΔE crumb (compared to C1)	-	17.66 ± 2.86 (b)	5.28 ± 3.39 (c)	8.92 ± 2.80 (bc)	13.05 ± 3.74 (b)	22.43 ± 2.71 (a)
ΔE crumb (compared to C2)	17.66 ± 2.86 (a)	-	14.74 ± 3.43 (ab)	9.60 ± 2.63 (b)	6.14 ± 2.52 (b)	5.69 ± 2.20 (b)
Water activity	0.951 ± 0.008 (a)	0.953 ± 0.009 (a)	0.938 ± 0.019 (a)	0.947 ± 0.023 (a)	0.940 ± 0.026 (a)	0.947 ± 0.019 (a)
Day of first microbial growth	6.00 ± 1.00 (b)	6.67 ± 0.58 (b)	6.00 ± 0.00 (b)	9.00 ± 1.00 (a)	6.33 ± 0.58 (b)	9.33 ± 0.58 (a)

**Table 4:** Starch, dietary fibre, protein, fat and moisture content of bread samples. Values represent the means ± standard deviation.

	Controls		Source of Fibre		High in Fibre	
	Baker’s Flour (C1)	Wholemeal Flour (C2)	EverVita FIBRA	EverVita PRO	EverVita FIBRA	EverVita PRO
Total starch (g/100 g)	40.56 ± 0.42 (a)	34.00 ± 1.10 (bc)	39.26 ± 1.18 (ab)	36.23 ± 2.19 (abc)	35.23 ± 1.05 (abc)	31.66 ± 2.16 (c)
Of which is digestible starch (g/100 g)	39.82 ± 0.02 (a)	33.17 ± 1.12 (bc)	38.63 ± 1.21 (ab)	35.51 ± 2.22 (abc)	34.51 ± 1.06 (abc)	31.03 ± 2.23 (c)
Of which is resistant starch (g/100 g)	0.74 ± 0.44 (a)	0.82 ± 0.02 (a)	0.63 ± 0.02 (a)	0.72 ± 0.03 (a)	0.72 ± 0.03 (a)	0.63 ± 0.07 (a)
Fibre (g/100 g)	2.1 *	4.8 *	3.8 *	3.5 *	6.6 *	6.5 *
Protein * (g/100 g)	9.5 *	8.4 *	9.7 *	10.1 *	10.1 *	11.5 *
Fat (g/100 g)	3.18 *	3.33 *	3.24 *	3.27 *	3.36 *	3.47 *
Moisture (g/100 g)	43.17 ± 0.19 (bc)	44.37 ± 0.80 (b)	42.04 ± 0.04 (c)	43.85 ± 0.12 (b)	43.20 ± 0.49 (bc)	46.68 ± 0.26 (a)

(*) based on calculation considering the information from the compositional analysis of the raw ingredients. Values with the same lower-case letter in a row do not differ significantly from each other.

## Data Availability

Not applicable.
